# Reconstructing community dynamics from limited observations

**DOI:** 10.1186/s40168-026-02449-y

**Published:** 2026-06-25

**Authors:** Chandler Ross, Ville Laitinen, Moein Khalighi, Jarkko Salojärvi, Willem M. de Vos, Guilhem Sommeria-Klein, Leo Lahti

**Affiliations:** 1 Department of Computing, University of Turku, Turku, 20014 Finland; 2 School of Biological Sciences, Nanyang Technological University, 50 Nanyang Avenue, Singapore, Singapore; 3 Organismal and Evolutionary Biology Research Programme, University of Helsinki, 00100 Helsinki, Finland; 4 Human Microbiome Research Program, Faculty of Medicine, University of Helsinki, Helsinki, 00100 Finland; 5 Laboratory of Microbiology, Wageningen University, Wageningen, The Netherlands; 6 Inria, University of Bordeaux, INRAE, F-33400 Talence, France

**Keywords:** Bistability, Exit time, Gaussian processes, Human gut microbiota, Microbial ecology, Stability landscape, Stochastic differential equation, Tipping points

## Abstract

**Background:**

Ecosystems tend to fluctuate around stable equilibria in response to internal dynamics and environmental factors. Occasionally, they enter an unstable tipping region and collapse into an alternative stable state. Being able to quantify and predict these dynamics is key to our understanding of how microbial communities vary over time and respond to perturbations.

**Results:**

Mechanistic models of microbial community dynamics often fail to characterise observed fluctuations in naturally occurring microbiomes and inform us about key dynamical properties such as stability and resilience. An alternative approach is to characterise the dynamical landscape using non-parametric models. However, the scarcity of long, dense time series data poses a severe bottleneck for characterising community dynamics using existing methods. We overcome this limitation by combining information across multiple short time series using Bayesian inference. By decomposing dynamics into deterministic and stochastic components using Gaussian process priors, we are able to predict stable and tipping regions along a unidimensional stability landscape while simultaneously addressing the associated uncertainty. In particular, we estimate a recently proposed probabilistic metric for resilience in multistable systems: the expected “exit time” out of the current stable state under stochastic fluctuations.

We validate our approach on simulated data and highlight in particular that our model is able to distinguish bistability from bimodality, which are often conflated in classical potential analyses. We further demonstrate our approach by re-analysing ecological time series data of lake cyanobacteria abundance, for which we recover similar results as a previous study using three orders of magnitude fewer data points. Finally, we use our model to re-evaluate the stability of previously proposed “tipping elements” in the human gut microbiota.

**Conclusions:**

We introduce a probabilistic non-parametric approach to characterise stationary community dynamics from short time series, which is potentially applicable to a broad range of systems in microbial ecology and beyond. We use this model to clarify the distinction between bistable and bimodal dynamics and to contribute to contemporary debates on the stability and resilience of ecological communities, in particular the human gut microbiota.

Video Abstract

**Supplementary information:**

The online version contains supplementary material available at https://doi.org/10.1186/s40168-026-02449-y.

## Introduction

Microbial communities are subject to stochastic dispersal, birth, and death processes [1], and incessant external perturbations caused by environmental fluctuations. Moreover, interactions between community members [2] or ecological memory [3] can generate complex dynamics [4–6], such as sudden shifts between alternative stable states characterised by different community compositions. Such multistable dynamics have been reported in diverse ecosystems ranging from freshwater plankton communities [7] to human gut microbiota [8–10]. Attempts have been made to disentangle these different ecological processes from time series [11]. However, the dynamics of ecological communities are notoriously difficult to model as soon as the community size exceeds a few species. This is especially true of microbial communities, due to the diversity and lability of interspecific interactions [4]. Thus, mechanistic models often fail to predict state shifts or to quantify key dynamical properties such as stability and resilience [12, 13].

An alternative approach is to characterise the system’s fluctuations statistically while making minimal assumptions about the underlying processes [14]. This has been used to detect early warnings from time series [15, 16] and to understand how systems change over time [17]. A popular approach is to assume that the dynamics are well characterised by a stationary probability density. This approach has been applied to various complex systems such as climate data [18, 19] and more recently microbial communities [20, 21]. However, these approaches have important shortcomings. They usually conflate the number of modes in the probability density with the number of stable states. This approximation is necessary when attempting to recover the dynamics from the probability density without access to time series data [9, 18, 20, 21]. Yet, it only holds under certain simplifying assumptions, and in particular, if the intensity of stochastic fluctuations does not vary with the system’s state [22]. Explicitly characterising the dynamics from time series, on the other hand, has thus far relied on long and dense time series in ecology [11, 14, 23]. Yet, even though the availability of time series data is quickly increasing in microbial ecology, the vast majority remain short and sparse due to practical or ethical constraints in sample collection—often no more than a few time points per sampling unit [9, 24].

In order to overcome these limitations, we propose a flexible probabilistic model to reconstruct complex system dynamics from multiple short time series. We show how the model can be used to detect a system’s stable and *tipping* regions, quantify multistability and resilience, and address uncertainty. We validate the model with simulated and real data. Finally, we highlight shortcomings in the currently popular techniques for the analysis of community stability and we use our approach to re-examine the stability properties of microbial taxa previously proposed to exhibit bistability: the so-called tipping elements of the human gut microbiota [9].

## Results

### Modelling approach

**Fig. 1:**
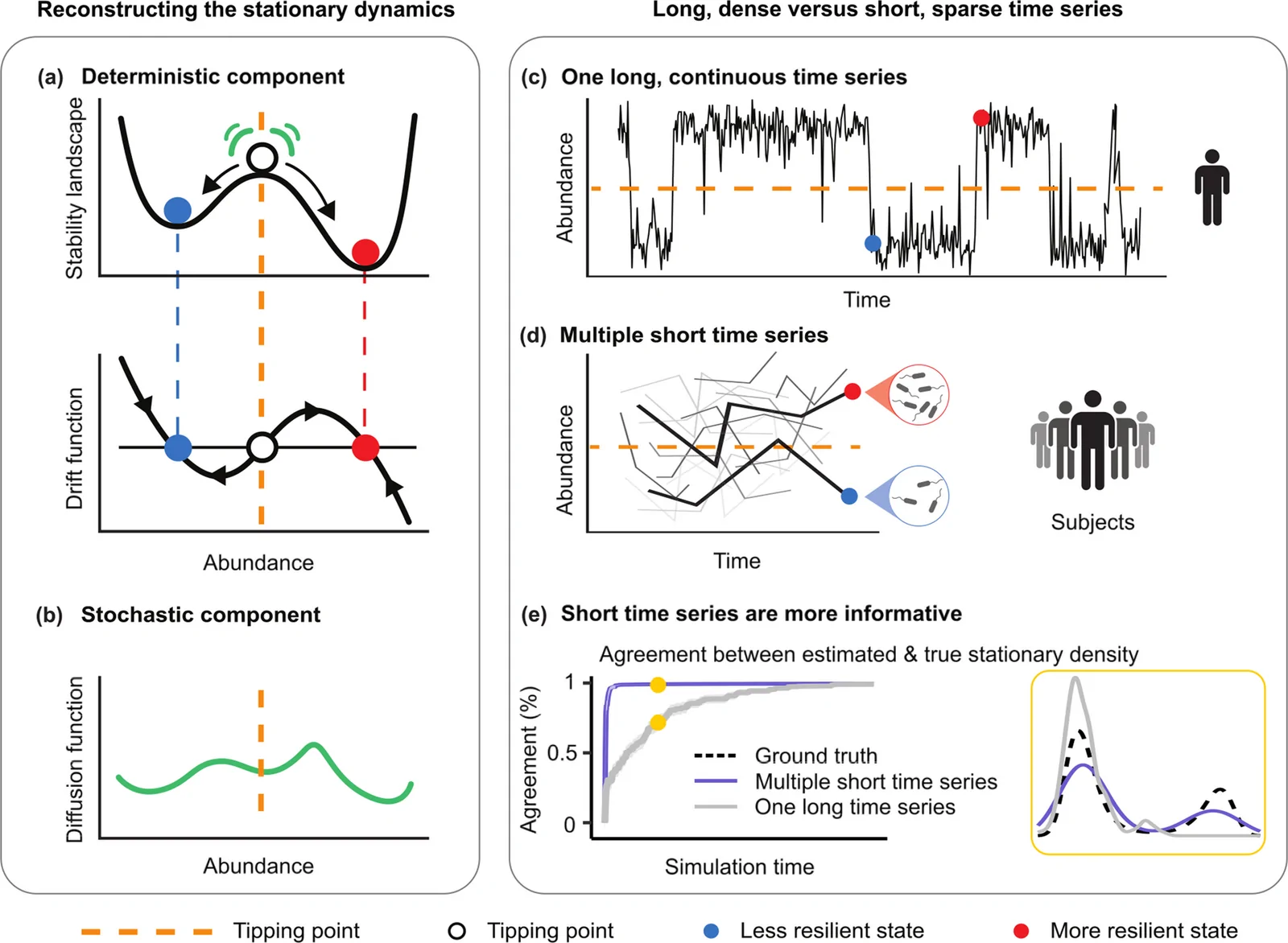
The stability landscape and key concepts. **a** A hypothetical bistable system with two stable states of low and high abundance, separated by an intermediate tipping point (dashed orange line). The dynamics can be described as movement along the stability landscape (upper plot) where at any given time, the system is likely to be found in either the less resilient state (blue) or the more resilient state (red, with a deeper valley) and less likely to be found in the unstable, middle tipping region. The drift function (lower plot) is the negative gradient of the stability landscape; its roots provide the location of the stable states and unstable tipping point of the stability landscape. **b** Movement along the landscape is subject to stochastic perturbations whose intensity may vary with abundance and is described by the diffusion function. **c** A hypothetical long and continuous series of observation from the system defined in **a**, **b** as assumed by most existing methods for dynamics characterisation. **d** A collection of short time series with the same underlying dynamics as in **c** more typical of the data encountered, for instance, in human gut microbiota research. **e** Short, independently sampled time series cover the range of possible measurements in a system more efficiently than a long, continuous time series of the same total length. Both curves show the average agreement with the ground truth stationary density over 50 independent simulations (see Methods). Two example densities calculated from the points highlighted in yellow are shown (in blue and grey) compared to the true stationary density of the process from which they were simulated (dashed black line, see also Additional file 2)

Our approach is based on the classical representation of ecological dynamics as movement along a stability landscape, also called potential landscape, caused by stochastic fluctuations (Fig. 1a, b) [14, 25]. The landscape is a function of the system state, quantified by an indicator variable such as the abundance of a key species, or a higher-level aggregate such as diversity or biomass. Univariate summaries are useful because stability landscapes are generally not well defined in multivariate systems [26]. In our applications, we defined the system state as the abundance of a focal bacterial taxon, after applying the CLR-transformation to the relative abundances in the case of the human gut microbiota data.

Local minima in the landscape represent stable equilibria, or “stable states”. Local maxima represent unstable “tipping points”, which mark the boundary between alternative basins of attraction. A lake may, for instance, exhibit distinct states of low and high cyanobacteria abundance, and fluctuate around one of the stable points until a large enough perturbation pushes the system across the tipping point, triggering a transition to the other state (Fig. 1a, b) [27]. Such transitions are sometimes described using the theoretical framework of bifurcations, where a gradual change in a control variable (nutrient input to the lake, for instance) shifts the stability landscape until the current equilibrium disappears, a point also commonly—and confusingly—referred to as the system’s “tipping point” in the literature [28]. Here, however, we assume the situation where no such control variable is available, so that all observations are described as following the same stability landscape. A control variable can be missing either because no individual variable has a significant influence on the system or because they could not be measured. In the latter case, the modelled stability landscape should be thought of as an effective landscape averaged across values of the control variable.

The dynamics can then be decomposed into a deterministic and a stochastic component, using a stochastic process model in which they are quantified by the drift and diffusion functions, respectively (Fig. 1a, b) [29]. Drift is the negative derivative of the stability landscape and encodes the shape of the landscape (Fig. 1a). The diffusion encodes the intensity of stochastic fluctuations as a function of the system state (Fig. 1b). Since the model operates on continuous state variables defined on the real axis, transformations may be required to map observed data into this domain. While the exact choice of transformation can affect the quantitative form of the inferred drift and diffusion functions, the qualitative dynamical features of interest, such as the presence and relative location of stable states and tipping points, should be preserved. Classical approaches to inferring this decomposition assume a specific shape for the drift and diffusion. This entails strong assumptions on the landscape shape, the number of possible stable states and fluctuation intensity along the landscape [18]. Alternatively, non-parametric models allow for a more flexible characterisation. However, existing inference methods for non-parametric models rely on computing the moments of the trajectory within bins of time points, which requires long and dense times series that are often not available [14, 19]. In contrast, we propose here a non-parametric approach based on Bayesian inference that allows us to leverage the collections of short time series common in ecological research. Another advantage of a probabilistic approach is that it quantifies uncertainty, which is essential in natural systems where stochasticity and measurement errors make it virtually impossible to determine the number of stable points with full confidence, or determine exact tipping point locations. These uncertainties should be presented as an integral part of the results. In particular, the *tipping region*, the interval with an elevated probability for state shifts, may be more useful to report than a tipping point.

Our approach builds on two key ideas. First, we guide the model with flexible Gaussian Process priors for drift and diffusion (Methods section), which were recently applied in microbiota research in other contexts [30, 31]. Second, assuming that all samples follow the same stationary dynamics, a collection of short, independently sampled time series can actually cover the state space more efficiently than a single long time series, as consecutive time steps often exhibit strong dependencies (Fig. 1c–e, see Additional file 2). As a result, our approach can use remarkably fewer observations than the commonly used alternative techniques.

**Fig. 2:**
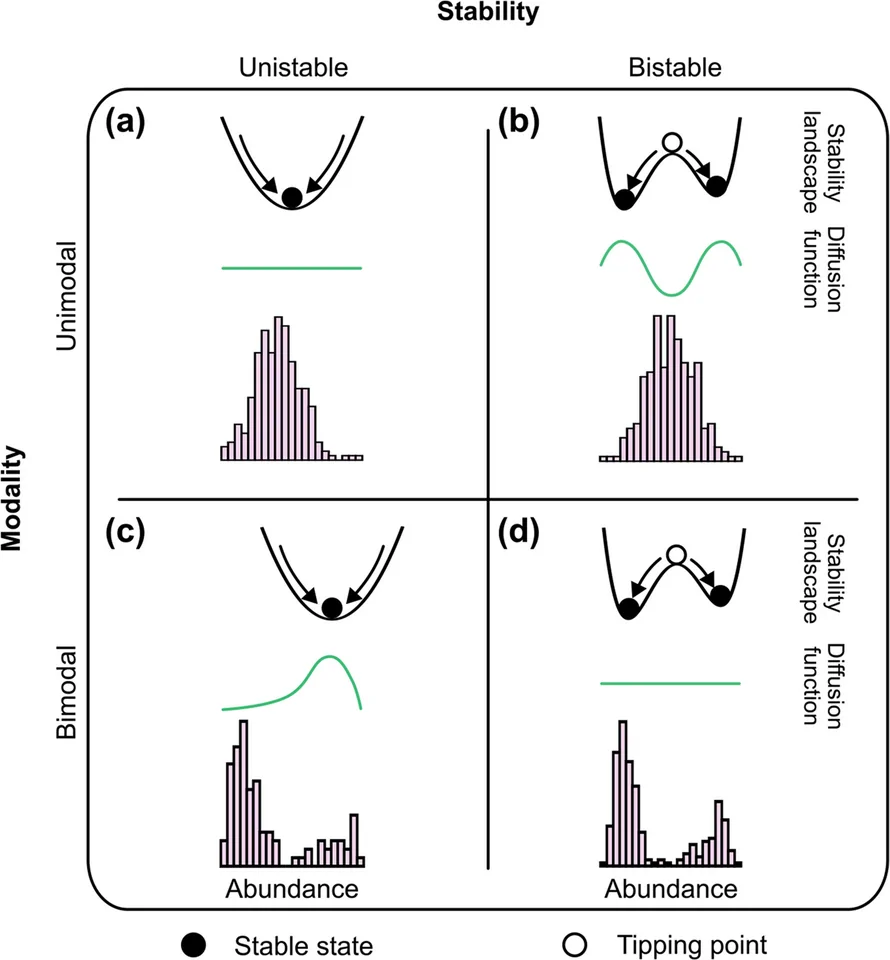
Disentangling modality and stability. **a**, **d** Usual cases where the bimodality of the data histogram coincides with the bistability of the underlying process: **a** the unistable landscape gives rise to a unimodal histogram and **d** the bistable landscape to a bimodal one. This is the case as long as the diffusion function (green curves) is relatively constant. **b**, **c** This congruency breaks in the presence of more complicated diffusion functions: with the illustrated diffusion functions, **b** the bistable potential gives rise to a unimodal histogram and **c** the unistable potential to a bimodal one

### Stability and modality

Multistable systems spend most of their time fluctuating around their stable points: this is reflected in the density of observations, which tend to be clustered around stable points. Accordingly, the “effective potential” obtained from the stationary distribution of observations is often used as an approximation for the stability landscape. For instance, the so-called “potential analysis” considers bimodal stationary density as diagnostic for bistable dynamics [18]. More generally, multimodality in the stationary density has frequently been treated interchangeably with multistability in the ecological literature [9, 18, 32].

The modes of the observed stationary density can be used to infer stability when the diffusion is approximately constant, that is, when the intensity of fluctuations does not depend on the system state (Fig. 2a, d). However, in general, the distribution of observations does not carry enough information to identify multistability. When stochastic fluctuations are wider close to the stable state than away from it, trajectories may occasionally escape toward regions of lower stability but reduced diffusion, which leads to prolonged residence times (Fig. 2c). As a result, state-dependent diffusion can give rise to multimodal abundance profiles even in the absence of attractors in the stability landscape (Fig. 2c). We term this scenario *diffusion-driven* bimodality, in contrast to the more classic *drift-driven* bimodality (Fig. S1). Conversely, in the case of a bistable system, increased fluctuation intensity around the stable states may similarly conceal the bistable dynamics behind unimodal observations (Fig. 2b). Increased fluctuation intensity around stable states may occur in ecological systems if the dynamics tend to accelerate around the system equilibrium, for instance because of increased growth rates and competition.

To assess whether state-dependent diffusion was appropriate to describe our datasets, we compared the predictive power on held-out data of a model with constant diffusion (that is, independent of the system’s state) to our flexible GP diffusion model. When modeling CLR-transformed abundance data, such as in our human gut microbiota application, a model with constant diffusion amounts to applying a multiplicative noise on species abundance, which is typical of environmental noise in ecology. We found that the flexible diffusion model consistently performed better as measured by the difference in the models’ expected log predictive densities (ELPDs), indicating that state-dependent stochasticity is clearly supported by the data (Fig. S2).

**Fig. 3:**
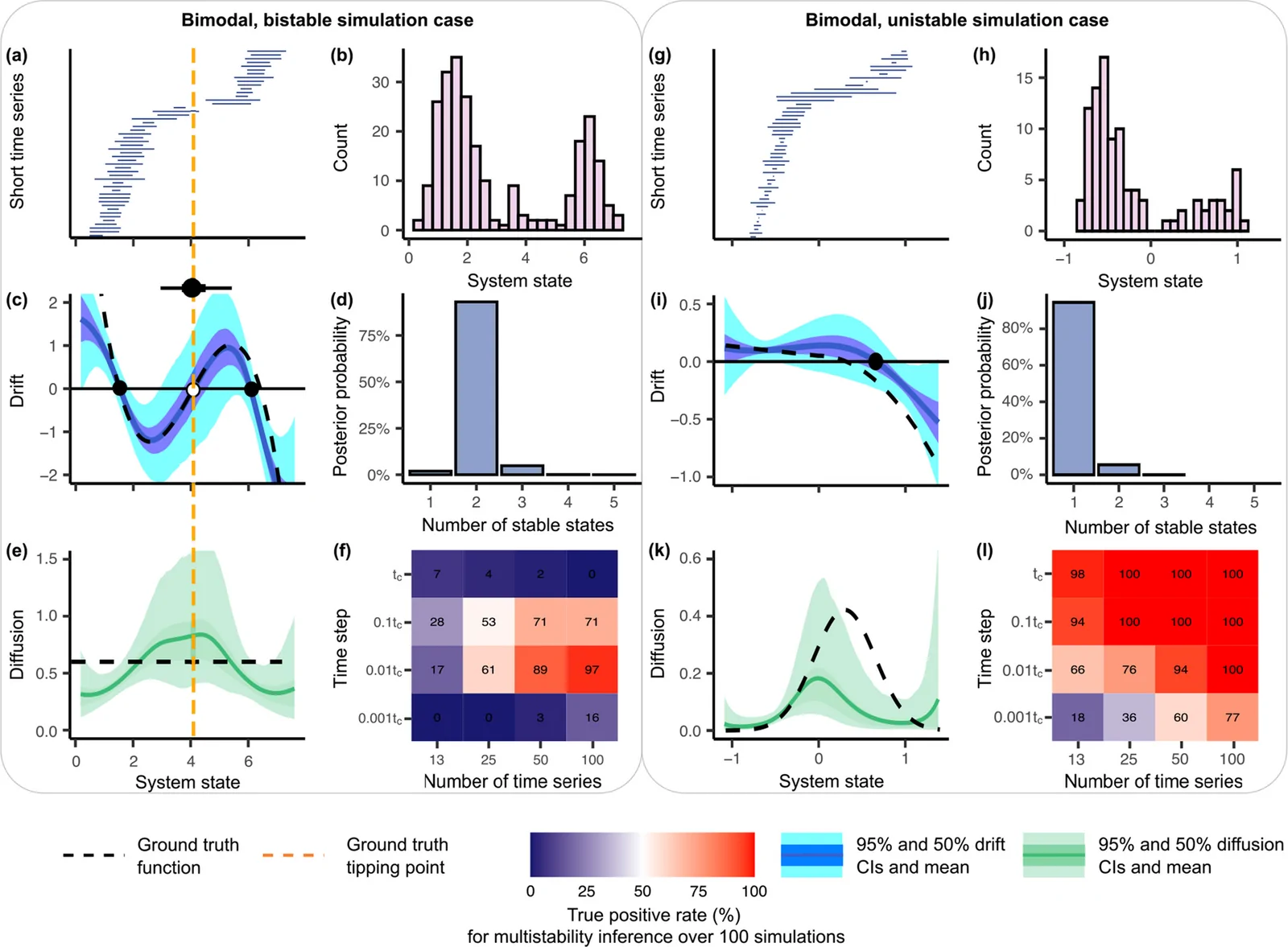
Inferring system dynamics and multistability. Here, system state is a one-dimensional state variable of the simulated system. **a**, **g** Short time series (5 points each in **a** and 2 in **g**) simulated from **a** a bistable stochastic process (cusp model), and **g** a unistable process. The known tipping point of the bistable process is represented by a dashed orange line. **b**, **h** Histograms of the short time series in **a**, **g**. **c**, **e**, **i**, **k** posterior estimates for the **c**, **i** drift and **e**, **k** diffusion functions, including the 50% (indigo and red, respectively) and 95% (cyan and salmon, respectively) credible intervals, and the ground truths (dashed black lines). Black and white points on the posterior mean of the drift function indicate stable modes and the tipping point, respectively. The posterior distribution of the tipping point—the tipping region—is summarised by its mean and 95% credible interval in the upper margin of **c**. **d**, **j** Posterior probability of the number of stable states. **f**, **l** Each cell represents the true positive rate (represented numerically within the cell and by its color) of detecting the stability of the underlying process in 100 independent simulations of 2-point time series, varying the number of time series (i.e. half the number of points) and the sampling time step defined as a fraction of the time scale $t_c$ of the process. Multistability inference is most accurate for intermediate sampling time steps, and generally improves with larger sample size

### Model assessment

We analysed model performance based on the well-established cusp simulation model [33], which is capable of generating a variety of uni- and bistable dynamics via a third-order polynomial drift and constant diffusion (Fig. 3a–f). We also generated bimodal but unistable dynamics with non-constant diffusion using a custom stochastic differential equation (Fig. 3g–l, Eq. 12 in the Methods section). In both cases, we simulated short time series and then reconstructed the drift and diffusion functions using our modelling approach. The true drift function and tipping and stable point locations retrieved by our model fell nearly entirely within the 95% posterior credible intervals (Fig. 3c, i). The diffusion generally followed the ground truth but exhibited larger deviations (Fig. 3e, k). More specifically, the diffusion follows the trend already reported in Arani et al. 2021 [14]: drift-diffusion models tend to underestimate the diffusion in the vicinity of stable points and overestimate it near the tipping point. A low level of fluctuations around stable points can be described either by a strong deterministic component or a weak stochastic one, and distinguishing between these is challenging from sparse data. Similarly, around the tipping point, a high level of fluctuations may be either attributed to a weak drift or a strong diffusion.

The two key factors affecting the performance of the model are the number of short time series and their density, quantified by the time step between two consecutive points (Fig. 3f, l). In general, more and denser time series improved inference. Indeed, shorter time steps are in better agreement with our discretisation approximation (see Eq. 2 in the Methods section). Nevertheless, inference accuracy dropped again for time steps that were too short to sample the state space efficiently, given the small number of time points per time series we consider (here, only two). We expressed the time step between two points as a fraction of $t_c$, a quantity we introduced to approximate the characteristic time scale of the dynamics from time series data before any model fitting. $t_c$ is defined independently of our model as the hypothetical time needed to traverse the apparent state space of the system through observed stochastic fluctuations (see Methods).

We found that inference of unistability from short and sparse time series is generally robust even when the data exhibit bimodal observation densities (Fig. 3l). Conversely, bistability can be detected with 71% to 89% reliability (true positive rate across 100 independent simulations) with as few as 50 time series with two points each for constant simulated diffusion, provided that the time step is on the order of 0.1$t_c$ or 0.01$t_c$ (Fig. 3f). Bistability inference is therefore robust to sparse data using our approach. Furthermore, if the time step is in the favourable range of about 0.01$t_c$ and there are at least 100 short time series, the drift and diffusion estimates are sufficiently accurate to permit the reliable inference of secondary quantities such as stationary densities and stable state resilience, as we describe in the next sections. Outside of this regime, insufficient temporal resolution relative to the intrinsic system dynamics can lead to biased drift estimates. Further analyses show that the accuracy of tipping point estimation closely mirrors the performance of multistability detection across the parameter space (Fig. S3): that is, performance improves with more time series and finer temporal resolution. The exit time stands out as particularly sensitive to small errors in the inferred tipping point and should therefore be interpreted with caution unless the time step falls in the favorable range of 0.1$t_c$ to 0.01$t_c$.

Remarkably, we found that the accurate inference of a bistable landscape does not require the direct observation of transitions between stable states, as illustrated by Fig. 3a, c. To further assess the effect of the directionality of observed state transitions on model inference, we simulated bistable time series and biased them by removing all state transitions from the high-abundance state towards the low-abundance state, but keeping state transitions in the opposite direction. We then compared model inference on these biased data with that on unbiased data with bidirectional state transitions, across 100 simulated datasets. We found that the inferred drifts and their equilibria (stable states and tipping points) were robust to this asymmetry in observed state transitions (Fig. S4). Hence, accurate inference depends primarily on state space coverage and the time scale of sampling, rather than on the number or direction of transitions, as shown in Fig. 3f. This property results from the prior distribution over the drift function, which strongly favors stationary dynamics across the state space, including in poorly sampled regions, as long as stationarity is supported by available data in better sampled regions (see the Methods section).

### Lake Mendota data

To illustrate our approach, we re-analysed the phycocyanin level time series data from Lake Mendota (WI, USA), a one-year dense time series of measurements made every few minutes over the year 2011 (Fig. S5a) [27]. Phycocyanin level is a proxy for Cyanobacteria abundance and used as a descriptor of the ecological state of the lake. We compared our approach to that proposed by Arani et al. (2021) [14] to analyse the same data. Arani et al. estimated the drift and diffusion for this dataset using a non-parametric drift-diffusion model similar to ours, therefore their approach can be considered the most directly comparable currently available in the ecological literature. However, they fitted their model by calculating the moments of the drift-diffusion stochastic differential equation (see Eq. 1 in the Methods section, Probabilistic model) using a single time series consisting of $6 \times 10^4$ equally-spaced data points (dt≈2.5 mins). In order to compare our approach to theirs, we selected 10 short time series (equally-spaced with dt=12 days between time series) of five equally-spaced time points each (dt=10 mins between adjacent points). We chose a 10-minute interval between observations, which corresponds to 0.005$t_c$, to match the time lag of 10 minutes that they applied in their analysis to avoid non-Markovianity. We found that our approach is capable of recovering, from these 50 observations only, a very similar dynamical landscape to the one they uncovered using three orders of magnitude more data points (Fig. S5a, b).

**Fig. 4:**
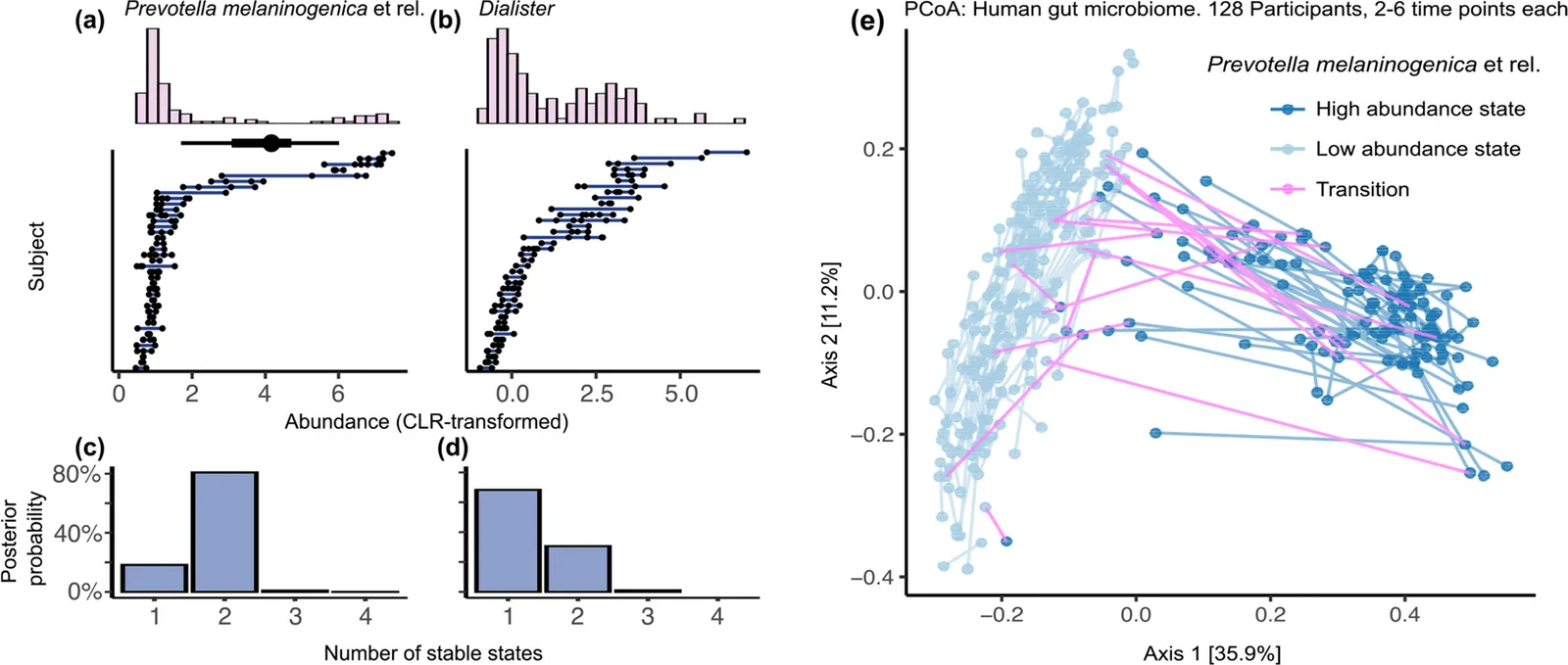
Reanalysis of human gut microbiota stability. **a**, **b** 39 short and dense time series of the abundance of (**a**) *Prevotella melaninogenica* et rel. and **b** *Dialister* (each collected from a different subject, with 2–6 time points) on which we ran our model, subsetted from the 128 original time series shown in **e** as described in Methods. The empirical histograms are shown in the upper plots, and the range of abundance variation for each subject in the lower plots. In **a**, the black horizontal bar summarises the posterior distribution of the tipping point location identified by the model (most probable location with 50% and 95% credible intervals). **c**, **d** Posterior probabilities for the number of stable states. **e** Ordination representing the overall community similarity (PCoA, Bray-Curtis index) between 358 human gut microbiota samples from 128 time series, including the subset of 39 dense time series shown in **a**, **b**. Dark and light blue points indicate the high and low abundance stable states of *Prevotella melaninogenica* et rel. identified by our model, respectively. The arrows represent the paths of individual subjects through time. Pink lines indicate transitions across the tipping point identified by our model

### Tipping elements

Recent population studies characterised the landscape of cross-sectional variation in gut microbiota composition (i.e. variation between individuals; [20]). Characterising the stability landscape has been more challenging due to the scarcity of population-level time series data and the lack of suitable methods applicable to limited time series. Our model is addressing this gap. Lahti et al. (2014) have previously reported evidence for the bistability of certain sub-communities of the human gut microbiota, the so-called *tipping elements* [9]. This analysis was based on taxonomic profiling of 1,006 western adults, however, only 78 of them had multiple time points ([9]). We extended this dataset by retrieving additional data for altogether 128 adults with short time series of 2–6 time points each (see Methods) [9, 34]. In order to respect the Euler-Maruyama approximation used in our approach while maintaining a sufficient number of time points, we included in the final model only those 39 individuals with time series that had sufficiently small time steps (between $0.01 t_c$ and $0.06 t_c$ depending on the taxon, Figs. 4a, b, S6, Methods).

Our drift-diffusion model identified bistability in 4 genus-level groups (out of the 62 groups with highest prevalence; see Methods and Fig. S6): *Bifidobacterium*, *Eubacterium biforme*, *Prevotella melaninogenica* et rel., and Uncultured *Mollicutes* (Fig. 4a, c and see Additional file 1: Fig. S7). The two stable states of *Prevotella melaninogenica* appear to be strong drivers of the overall community state as they clearly structure the landscape of community-level variation across individuals (first two PCoA axes, Fig. 4e). This is consistent with the *Prevotella* genus being a key driver of the five identified enterosignatures of the human gut microbiome [35]. Nevertheless, when we examined pairwise relationships between taxa identified as bistable, we noticed signs of weak coupling and partial exclusion, but no strong signal of coordinated transitions across the taxa (Fig. S8). This suggests that the observed dynamics do not reflect a fully shared attractor structure between individual taxa [36–38], in line with the concept of tipping elements. However, the previously reported tipping elements *Bacteroides fragilis* et rel., *Dialister*, and *Uncultured Clostridiales* were deemed unistable by our model, despite bimodal abundances and in contrast to the evidence for bistability originally reported using a cruder approach on a subset of the same data [9] (Fig. 4b, d and see Additional file 1: Fig. S9).

**Fig. 5:**
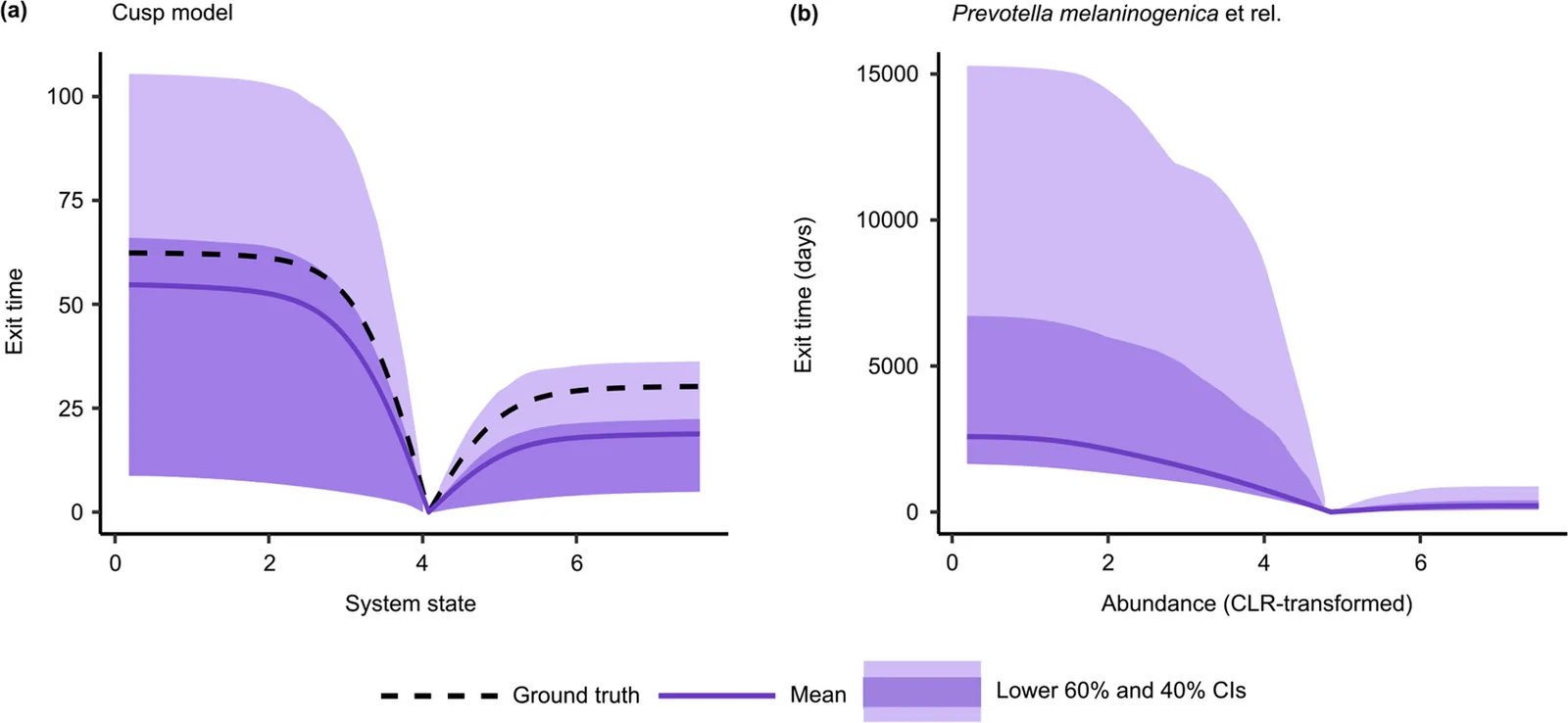
Predicted exit time. **a** Exit times predicted as a function of abundance, calculated from the simulated short time series shown in Fig. 3a. The dashed black line represents the known ground truth. **b** Exit times predicted for the *Prevotella melaninogenica* et rel. data of Fig. 4a. In (**a**) and (**b**), the posterior distributions are computed conditionally on the the location of the tipping point, and are represented by their mean (dark purple line) and their 40% and 60% lower credible intervals (dark and light purple regions, which are bounded by the posterior draw with the smallest exit time value)

### Resilience and exit times

Resilience quantifies the system’s tendency to remain in its current state in the face of perturbations [39]. Recently, Arani et al. (2021) [14] proposed the expected exit time as a new measure of resilience under continuous stochastic perturbations. This metric quantifies how long the system is expected to remain in its current stable state given its observed fluctuation regime [40]. Conveniently, the exit time can be directly derived from the inferred drift and diffusion functions [40]. The explicit incorporation of stochastic variation sets this measure apart from other measures of resilience. However, the original formulation relied on long and dense time series and the exit time itself is sensitive to small differences in the drift, diffusion, and tipping point estimates. Here, we provide a probabilistic estimate of exit time, which accounts for the uncertainty arising from limited observations (see the Methods section). Our approach could reliably estimate the exit time as a function of system state from the same simulated short time series as in Fig. 3a (Fig. 5a). Furthermore, the estimated exit times for the bistable *Prevotella melaninogenica* et rel. are in line with the time scales observed in the data (Fig. 5b, see Additional file 1: Fig. S10). However, the calculation of the exit time and its uncertainty are sensitive to the ratio between drift and diffusion and to the tipping point location. When the diffusion is low compared to the drift, the dynamics become strongly deterministic and the exit time may rise dramatically as a result. One avenue for future development is to bound the exit time by better incorporating it into the Bayesian model formulation through a prior distribution that would limit the space of possible functions. Designing such a prior using Gaussian Processes is not trivial, however, as it needs to verify three boundary conditions imposed by the definition of the exit time [41] (see Methods).

## Discussion

Observed population frequencies have been commonly used to characterise alternative states in ecological communities and other complex systems [18, 20]. Despite the recent popularity of this approach, the influence of stochasticity cannot be reliably detected merely from static observation frequencies without making strong assumptions on the distribution of stochastic fluctuations. Access to time series makes it possible to disentangle the stochastic and deterministic components in more general scenarios. However, quantifying the stability landscape with scarce time series data remains challenging in many real applications. We introduced a flexible method for reconstructing the stability landscape from limited observations. Our model quantifies changes in the drift and diffusion along the landscape, which permits the description of essential dynamical properties. Validation with real and simulated data demonstrate that the model can reliably locate stable equilibria and tipping regions, detect multistability using three orders of magnitude fewer points than another recent approach [14], and estimate exit times. Our simulations indicate that the inference of these quantities remains robust even when observed data exhibit no transition between stable states or asymmetric transitions. Related versatile models have been used to model thermal fluctuations in small particles in fluids [42], consumer behaviour [43], stocks [44], and decision outcomes in neuroscience [45]. Even though Gaussian Process regularisation has been leveraged to model financial and climatic data (e.g. [19]), to our knowledge such models have not yet been developed in microbial ecology.

The major limiting factor for model inference is the temporal resolution of the data, which should be high enough relative to the intrinsic dynamics of the system. We introduce a metric, the apparent characteristic time scale of the dynamics, that is readily calculated from time series data as a means to assess the suitability of the data. We demonstrate the use of this metric by subsetting our human gut microbiota data accordingly before proceeding to model inference. The requirement on temporal resolution should also be balanced with that of having sufficiently many independent observations to ensure adequate coverage of the state-space. Regions that are sparsely sampled make estimates of tipping points and stable states less reliable or cause them to be missed altogether. These limitations propagate to derived quantities such as the exit time, which requires bistability (or higher-order stability) to be calculated and is sensitive to the inferred location of the tipping point.

Our work demonstrates the utility of a probabilistic approach to the joint analysis of multiple time series. Nevertheless, one of the key limitations is the assumption that all observed short time series follow similar underlying dynamics. This limiting assumption could be relaxed by hierarchical extensions that incorporate individual deviations. Moreover, our model assumes that the overall dynamics does not change over time. Thus, changes in the stability landscape [28] or higher-order effects such as ecological memory [3] might set additional challenges. Time-varying models, or models where the landscape shape is governed by a control variable, could be a way forward, since our model cannot currently be applied to systems that undergo regime shifts and distinguish them from a fixed stability landscape with coexisting stable states. However, the added computational complexity of such models is a key limitation for Gaussian Processes in large datasets.

Our approach is flexible enough to accommodate many different forms of drift and diffusion, including the skewed and multistable stability landscapes characteristic of microbial communities [9]. Although the role of stochasticity on the shape of stationary densities has been widely ignored until recently [32], the importance of distinguishing between modality and stability in complex systems is being increasingly recognised in the literature [22, 46, 47]. We emphasise here non-constant diffusion as a key driver for the incongruency between stability and modality, but this phenomenon could also be attributed to other dynamical properties, such as non-normality or the presence of memory in the distribution of stochastic fluctuations [22]. Our approach could possibly be extended to account for these additional factors. Ecologically, we interpret our flexible noise function to be an effective, aggregated representation of multiple sources of stochasticity including demographic noise, environmental fluctuations, and other unobserved processes. Just as species interactions and growth rates depend on relative abundances and community composition [48, 49], the magnitude and sources of stochasticity are also expected to vary across the state space in non-linear ways. As a result, a state-dependent diffusion term can capture heteroskedasticity in ecological dynamics, rather than corresponding to a single mechanistic process.

In the context of microbial ecology, our approach models the stability landscape of each bacterial taxon independently, which can be interpreted as a one-dimensional projection of higher-dimensional community dynamics. It is common for a key taxon to disproportionately shape the structure of the community, so that the system effectively behaves largely univariately: this is the case of Cyanobacteria in Lake Mendota, *Prevotella melaninogenica* et rel. in our healthy human gut microbiota dataset (Fig. 4e), or *Clostridioides difficile* in highly dysbiotic microbiomes [50]. Nevertheless, bistability exhibited by individual taxa is not a direct indication of alternative stable states at the community level, nor does the absence of bistability in individual taxa exclude the possibility of bistability at the community level, which may arise from combinations of states across taxa. Moreover, combinations of bistable taxa or even a single taxon exhibiting bistability could lead to multiple emergent stable states at the community level. Such hypotheses could only be probed more deeply with a multivariate approach, which represents a promising future development of our framework.

This work addresses a timely challenge in areas such as human microbiota research, where long and dense time series are not commonly available and the system presents a mixture of overlapping and often poorly described processes. It also provides new insights into the modelling of complex ecosystem dynamics. In particular, the probabilistic approach extends the point-wise concepts of stable and tipping points to broader stable and tipping regions. This can naturally accommodate the uncertainty and fluctuations that are characteristic of many real systems [14]. Our non-parametric approach does not require information on the underlying mechanisms, and is as such potentially applicable to a broad range of complex systems beyond microbial ecology.

## Methods

### Probabilistic model

Our approach employs a drift-diffusion stochastic differential equation model, which assumes that the stationary dynamics can be decomposed into deterministic and stochastic components [51]. We assume an underlying stochastic differential equation for the process, where the dynamics follow a deterministic drift function but are perturbed from this path by a stochastic diffusion function. The mathematical representation of this is given by the Langevin equation [52]

1 $$\begin{aligned} \textrm{d}x(t) = f(x(t))\textrm{d}t + \sqrt{g(x(t))}\textrm{d}W(t), \end{aligned}$$

where *x*(*t*) is the process being modelled at time *t*, *f* is the drift, *g*/2 is the diffusion, and *W* is the Wiener process, i.e. white Gaussian noise. Following the Euler-Maruyama scheme, which we know to be convergent [53], we can discretise Eq. 1 so that

2 $$\begin{aligned} \Delta x \simeq f(x_n)\Delta t + \sqrt{g(x_n)}\Delta W, \end{aligned}$$

where $\Delta W = W_{n+1} - W_n$ is normally distributed according to $\mathcal {N}(0, \sqrt{t})$. This step also enforces the assumption of Markovianity in the model. Since the first term is deterministic, we can equally think of Δx as being normally distributed:

3 $$\begin{aligned} \Delta x \sim \mathcal {N}\left( f(x_n)\Delta t, \sqrt{g(x_n)\cdot \Delta t}\right) . \end{aligned}$$

Equation 3 acts as the likelihood in our Bayesian framework.

### Gaussian processes

Since these data tend to be very noisy, and stochastic differential equations (SDEs) are governed by two functions whose forms are unknown, they pose a problem for parametric models. Gaussian processes (GPs) act as non-parametric priors in that they do not assume any particular underlying functional form. Instead, they are entirely characterised by their mean and covariance functions, capable of encoding properties such as smoothness and periodicity. They are also powerful in the sense that we can use them for predictive inference: we can query the function’s value at points where we do not have observations. We model both the drift and diffusion as flexible functions with Gaussian process priors, allowing them to capture complex state-dependent patterns smoothly and continuously. Their main downside is that they scale as $\mathcal {O}(n^3)$, where *n* is the number of observations, and, as a result, they are not ideal for large datasets (more than a few hundred points). They act as priors over functions, and are denoted

4 $$\begin{aligned} \boldsymbol{\phi } (\textbf{x}) \sim \mathcal{G}\mathcal{P}(\textbf{m}(\textbf{x}), \textbf{K}(\textbf{x},\mathbf {x'})), \end{aligned}$$

where the $\mathcal{G}\mathcal{P}$ notation for the distribution indicates that this is an infinite-dimensional extension of the multivariate normal distribution such that any finite set of observations $\textbf{x}_i \in \mathbb {R}^D$ will result in a vector of values $\boldsymbol{\phi }(\textbf{x}) = (\phi (\textbf{x}_1), \phi (\textbf{x}_2), ... \phi (\textbf{x}_D))$ that are jointly Gaussian. $\boldsymbol{\phi }(\textbf{x})$ is the vector of the function $\phi : \mathbb {R}^D \rightarrow \mathbb {R}$, $D\in \mathbb {N}$, evaluated at each of the observations $\textbf{x}_i$, $\textbf{m}(\textbf{x})$ denotes the mean function evaluated over all observations, and $\textbf{K}(\textbf{x},\mathbf {x'})$ is the covariance between all possible pairs of observations. We set the mean function to zero.

The Gaussian process kernel $\textbf{K}$ is a positive semi-definite function that holds the majority of our prior beliefs about the function we want to model. We set the kernel for drift prior to be the sum of an exponentiated quadratic kernel and a linear kernel, that is

5 $$\begin{aligned} \textbf{K}_f(x, x') = \sigma _q^2\textrm{exp}\left( -\frac{1}{2l^2}(x-x')^2\right) + \sigma _b^2 + \sigma _l^2(x-c)(x'-c). \end{aligned}$$

The $\sigma _q$, $\sigma _b$, and $\sigma _l$ terms in Eq. 5 are variance parameters, *l* is the length scale, and *c* is a centring parameter. The exponentiated quadratic kernel (first term in Eq. 5) leads to functions with the desirable property of infinite differentiability. The linear kernel (second and third terms in Eq. 5) will cause the drift, in a region where there is no data point, to tend towards a line with a slope close to what it had in the adjacent region with data. Its inclusion aids in producing the desired end behaviour: instead of going toward zero, we want the drift to approach plus or minus infinity, which is required if we are to obtain stationary dynamics. The diffusion on the other hand only benefits from the properties of the exponentiated quadratic kernel since we cannot say anything a priori about its end behaviour. The one quality we do require that the diffusion have, however, is non-negativity. It is not straightforward to enforce this in the Gaussian process prior itself. In order to constrain the model, we use the following sampling statement for Eq. 3:

6 $$\begin{aligned} \Delta x \sim \mathcal {N}\left( f(x_n)\Delta t, \sqrt{\textrm{exp}(\hat{g}(x_n))\cdot \Delta t}\right) , \end{aligned}$$

where $\textrm{exp}(\hat{g}(x_n)) = g(x_n)$. In practice, we set a Gaussian process prior on $\hat{g}(x_n)$ rather than $g(x_n)$, and then transform it back to the actual quantity of interest $g(x_n)$.

### Model inference

We base model inference on a number of assumptions. We are concerned here with datasets that comprise several short times series that come from different but related sampling units, such as different study participants in the case of human gut microbiota. These data are assumed to be a one-dimensional description of the system and to sufficiently cover its state-space. To pool information between limited data, we assume that they represent different realisations from the same (or similar) underlying dynamics, and model them jointly. We assume that the first two moments of the stochastic differential equation, the drift and the diffusion, are sufficient in describing the dynamics. In addition, we assume that the time series are stationary, Markovian, and that the data takes sufficiently small time steps to minimise the error attributed by the Euler-Maruyama discretisation.

We set standard Inverse-Gamma(2, 2) priors on all variance parameters and Inverse-Gamma(5, 5) priors on length scale parameters. Inverse-gamma distributions are appropriate here as they are defined on the positive real line, suppress unrealistically small values while retaining heavy tails that allow for larger values, and concentrate prior mass over reasonable ranges. As such, they act as weakly informative priors. In terms of the length scale parameters, this allows for either curvy, high frequency functions or practically linear, low frequency functions, depending on the data. This behaviour is desirable in both the drift and diffusion functions. If the system is bimodal but not bistable, a flexible diffusion prior is needed. Conversely, if it is bistable, a flexible drift prior is needed. The linear and exponentiated quadratic kernels’ variance priors are the same because, in some cases, they are given equal weight. In the case of a bistable drift, the exponentiated quadratic kernel’s learned variance should have a greater influence. On the other hand, in the case of a unistable drift, the linear kernel’s variance should take the dominant role.

We performed Bayesian inference using the probabilistic programming language Stan, which uses Hamiltonian Markov chain Monte Carlo. We evaluated the convergence of the model using the statistics available in Stan, namely, the Rhat and n_eff values. In all cases, we used 4 chains with 2000 iterations per chain.

### Characteristic time scale

The time series need to be sufficiently dense to satisfy our modelling assumptions. To assess this, we introduce a time scale that can be calculated from any time series data, provided it can be assumed to follow approximately stationary dynamics. We define this time scale as the apparent time needed to traverse the observed range *d* of the data through stochastic fluctuations. We expressed this condition as $\sqrt{\langle \Delta x ^2 / \Delta t \rangle \cdot t_c} = d$, where $\langle \Delta x ^2 / \Delta t \rangle$ is the average of $(x(t+\Delta t) - x(t))^2 / \Delta t$ over the time series, which yields $t_c = d^2/ \langle \Delta x ^2 / \Delta t \rangle$. We expressed all time steps used in this paper as a fraction of this time scale. In the context of our model, $\langle \Delta x ^2 / \Delta t \rangle$ can be seen as an approximation for $\langle g(x)\rangle$ since $g(x) = \lim _{\Delta t \rightarrow 0}\frac{1}{\Delta t}\mathbb {E}[(x(t+\Delta t) - x(t))^2]$ [54]. We use $t_c$ as a practical diagnostic for assessing whether the temporal resolution of the data is compatible with the assumptions of the model.

### Derived quantities

For our purposes, the quantities of interest that can be derived directly from the drift and diffusion functions are the stationary density, stability landscape, multistability, and exit time.

The stationary density tells us how likely we are to find the system in a given state. Given the drift and diffusion (1), it is possible to derive the stationary distribution via [55]

7 $$\begin{aligned} \pi (x) \propto \frac{1}{g(x)}\textrm{exp}\left\{ 2\int _{x_0}^{x}\frac{f(y)}{g(y)}\textrm{d}y\right\} , \end{aligned}$$

where $x_0$ is any point in the state space of *x* and the constant of proportionality is the normalisation factor. The effective potential equivalently summarises the stationary state and is given by

8 $$\begin{aligned} U_{\textrm{eff}}(x) = -\textrm{log}\ \pi (x). \end{aligned}$$

The stability landscape is related to the drift via the transformation ([56]):

9 $$\begin{aligned} U(x) = -\int _{x_0}^{x}f(y)\textrm{d}y \end{aligned}$$

and reflects the underlying stability of the system.

The multistability posterior reflects the model’s confidence in the number of stable states underpinning the dynamics (Figs. 3d, j and 4c, d). The minimum number of stable states is one, and the posterior probabilities of the number states supported by the model sum to 100 %. When calculating this quantity, we looked at the roots of the posterior drift draws with positive slopes (tipping points) and negative slopes (stable points). In order to obtain a stationary solution, we required that the number of stable equilibria be one more than the number of tipping points in the system. Any draws that did not meet this requirement were excluded from the calculation since classifying their stability would be ambiguous. For each of the remaining draws, we could calculate the posterior of the multistability of the system by counting the number of roots with a negative slope.

Finally, the mean exit time can be determined by solving the second order ordinary differential equation:

10 $$\begin{aligned} f(x)\frac{\partial T(x)}{\partial x} + \frac{g(x)}{2}\frac{\partial ^2T(x)}{\partial x^2} = -1, \end{aligned}$$

where *T*(*x*) is the exit time [14]. In order to solve (10), we need to impose three boundary conditions: the slope must tend to zero as the state variable approaches $\pm \infty$ and the exit time must be zero at the tipping point. We achieved this using a custom finite difference method to numerically approximate the solution.

### Exit time uncertainty estimation

After running the model and obtaining posteriors for the drift and diffusion functions, we can calculate the exit time posterior by computing (10) over each of the posterior draws. The resulting exit time posterior draws can have vastly different tipping point locations which makes it hard to visualise. In order to quantify the uncertainty, we pruned the posterior draws to retain only those draws that had the same tipping point (up to the discretisation step) as the posterior means of the drift and diffusion. Then, starting from the draws with the smallest exit time values and working up, we calculate the lower 60% and 40% credible intervals. In this way, we avoid the draws with extremely large values which would be captured with standard credible intervals and we obtain good lower bound estimates on the exit time for a given tipping point.

### Simulation model

The model was tested on the well-studied stochastic process called the cusp catastrophe model. All of its dynamical properties such as its drift, diffusion, stationary density, and so on have analytical forms for comparison [56]. Not only do we know the ground truth, but the model allowed us to test a wide range of drift topologies including those that lead to unistable, bistable, and skewed stationary densities. Those that we expect to find in real data are not limited to any specific case. In particular, it is a stochastic process of the form

11 $$\begin{aligned} \textrm{d}x(t) = r(\alpha + \beta (x(t)-\lambda ) - (x(t)-\lambda )^3)\textrm{d}t + \sqrt{\epsilon }\textrm{d}W(t), \end{aligned}$$

where $\alpha , \beta , \lambda \in \mathbb {R}$, and r,ϵ>0 are the free cusp parameters.

Short time series are generated by drawing a starting value from the stationary density of the cusp model which is analytically known using inverse transform sampling. We then evolve the system using Eq. 11 and the Euler-Maruyama approximation scheme with time steps of 0.01 in order to obtain a convergent simulation. These high resolution time series are then subsetted to the desired time step that is larger than the 0.01 resolution. This process was repeated for each short time series.

For the bimodal, unistable case, we used custom drift and diffusion functions and evolved them according to the Euler-Maruyama scheme, sampling the starting points from the approximate stationary density in the same fashion as before. We chose the drift and diffusion as

12 $$\begin{aligned} f(x) = \left\{ \begin{array}{ll} \textrm{exp}(-0.08x) - 0.95 & x\le 0 \\ -0.5x^2+0.05 & x> 0 \\ \end{array}\right. \quad \text {and}\quad g(x) = 0.844\cdot \textrm{exp}[-(2x-0.6)^2]. \end{aligned}$$

### Short time series

In order to demonstrate the efficacy of short time series in covering the state space of a system, we simulated 50 independent short and long time series from the cusp model (above), each with the same total simulation time of 250. Applying an expanding window approach to these time series starting from a total simulation time of 1 and increasing by increments of 1, we calculated the Kullback-Leibler divergence between the ground truth stationary density of the system and the density approximated from the data histograms. We then normalised the results and subtracted them from 1 to obtain our agreement measure (Fig. 1e, see Additional file 2). In general, the histograms generated from the short time series converged much more rapidly than those of the long time series. In multistable systems, sampling times must be on the order of the characteristic time scale of the system (see above, characteristic time scale) to observe the alternative stable states of the system in continuous time series. Independently sampled short time series, on the other hand, are likely to sample all stable states on any timescale.

### Gut microbiota profiling

We retrieved a targeted subset of previously collected human gut microbiota profiling data from the HITChip Atlas database maintained at the Wageningen University and Research Center [9, 34]. In summary, DNA from faecal samples was extracted via the repeated bead beating (RBB) method and hybridised on the short oligos on the HITChip microarray [57], which has been designed to quantify the relative abundance of 130 genus-level groups that collectively capture the majority of the human gut microbiota variation. This array-based profiling technique provides an alternative to the current sequencing-based profiling techniques with high reproducibility [57]. To work with compositional data, we applied the standard centred log-ratio transformation (CLR) [58]. We excluded individuals with reported health issues, severe obesity, antibiotic use, and interventions, obtaining data from altogether 128 adults who had data from two or more time points. For the drift-diffusion model (Methods), we additionally excluded time steps of more than 45 days, leaving 39 subjects for dynamical analysis (Fig. 4a, b); this corresponds to time steps in the range of $0.01\cdot t_c - 0.06\cdot t_c$ (Fig. 3f, see “Characteristic time scale” above). We analysed the 62 most prevalent genus-level groups showing at least 20% prevalence (excluding *Prevotella oralis* as it was strongly covarying with *Prevotella melaninogenica*, possibly because of a methodological artefact).

## Supplementary information

Additional file 1: Figure S1. An alternative representation of the distinction between modality and stability. Figure S2. Comparing the performance between a constant diffusion model and our flexible diffusion model. Figure S3. Dependence of tipping point inference on the number of short time series and time step. Figure S4. Model performance when transitions unidirectional versus bidirectional. Figure S5. Re-analysis of Lake Mendota data with fewer time points. Figure S6. A table of all systems studied with our model. Figure S7. Four taxa in the HITChip data set that show evidence of bistability. Figure S8. The abundances of bistable taxa plotted against each other. Figure S9. Five taxa identified as tipping elements in Lahti et al. (2014). Figure S10. Transition time scales present in the gut microbiota.

Additional file 2. A video simulating how short time series approximate the true underlying stationary density of a process more efficiently than a single, long continuous time series.

## Data Availability

The code and data needed to reproduce the contents of this article are available with a Zenodo DOI at the following link: https://doi.org/10.5281/zenodo.14989142. The code was written in R version 4.1.0 and rstan_2.21.2 [59].
