# Supplementary material for: Reconstructing community dynamics from limited observations
Source: Microbiome. 2026 Jun 25;14:209. doi: 10.1186/s40168-026-02449-y (PMC13540962; doi:10.1186/s40168-026-02449-y)
Supplement: Supplementary file 1 [file 40168_2026_2449_MOESM1_ESM.pdf]

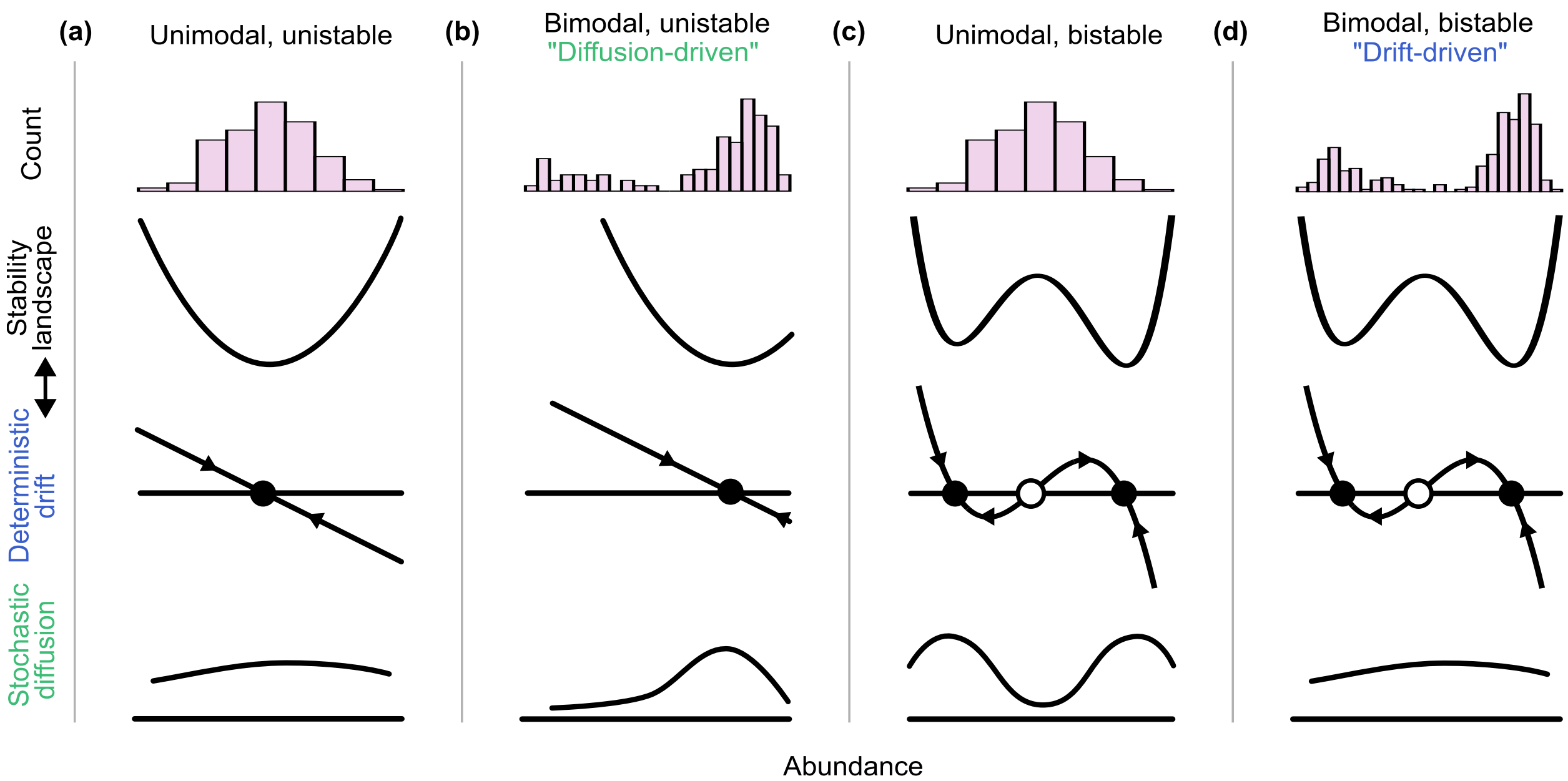

Figure S1 An alternative representation of the distinction between modality and stability. The drift function quantifies the stability landscape but diffusion plays a key role in the type of data we observe. (a) Unistable drift with approximately constant diffusion can generate unimodal stationary densities. (b) Unistable drift and non-constant diffusion can generate "diffusion-driven" bimodal stationary densities. (c) Bistable drift with highly variable diffusion can generate unimodal stationary densities. (d) Bistable drift, combined with approximately constant diffusion generates "drift-driven" bimodal stationary densities.

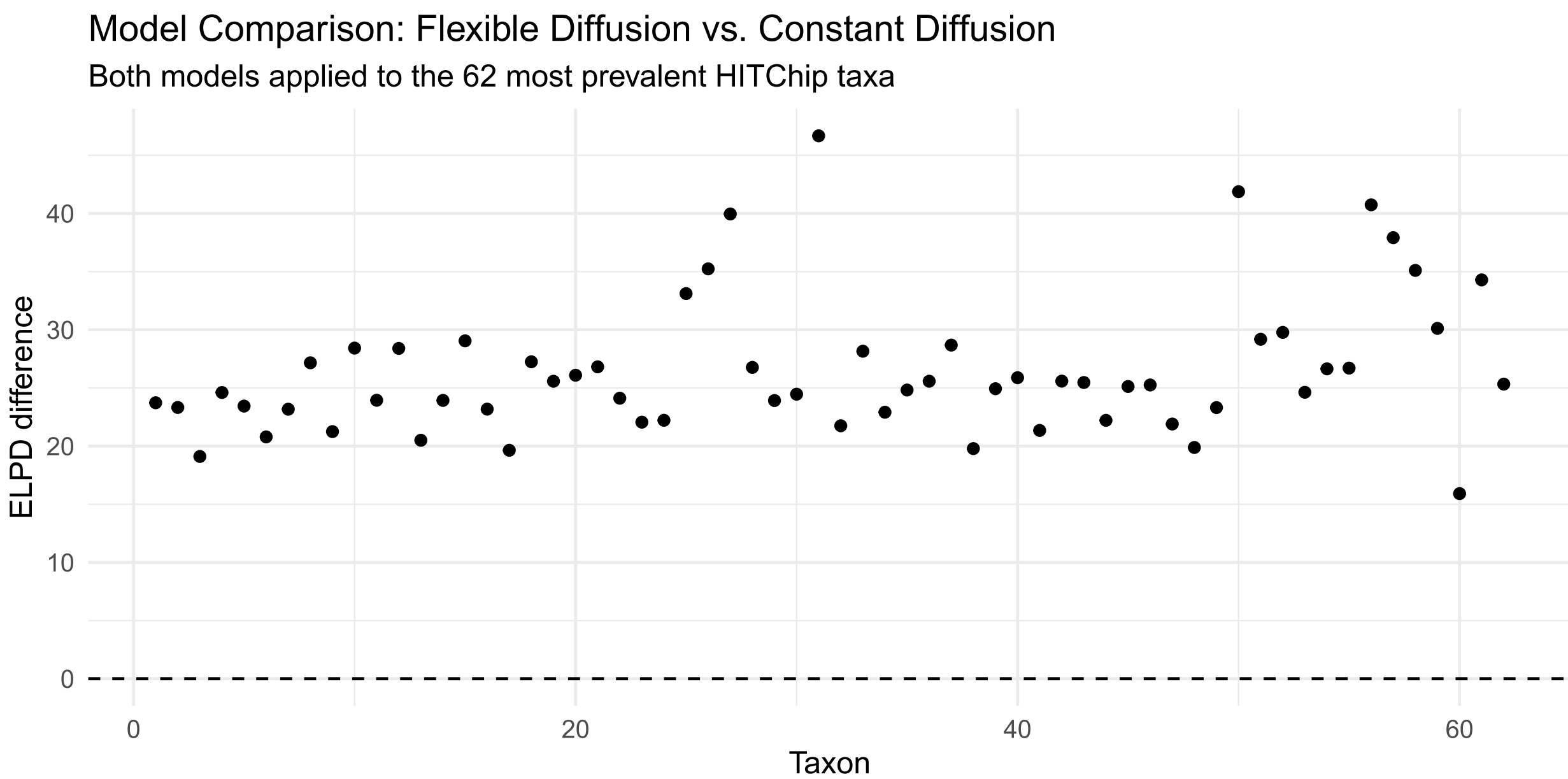

Figure S2 ELPD is the theoretical expected log pointwise predictive probability density when a model is exposed to a new dataset. We approximated this quantity using the "loo()" function from the "loo" package in R which approximates leave-one-out (LOO) cross-validation using Pareto smoothed importance sampling (PSIS). We compared, for the 62 most prevalent HITChip taxa, our model with a flexible diffusion function parametrised by a Gaussian process prior to a model with a constant diffusion function. In this case, leave-one-out refers to removing one subject's time series. For each taxon, we report the difference in ELPD values between the two models. We find that the flexible diffusion model consistently outperforms the constant diffusion model when faced with unseen data, indicating that state-dependent stochasticity is strongly supported by the data.

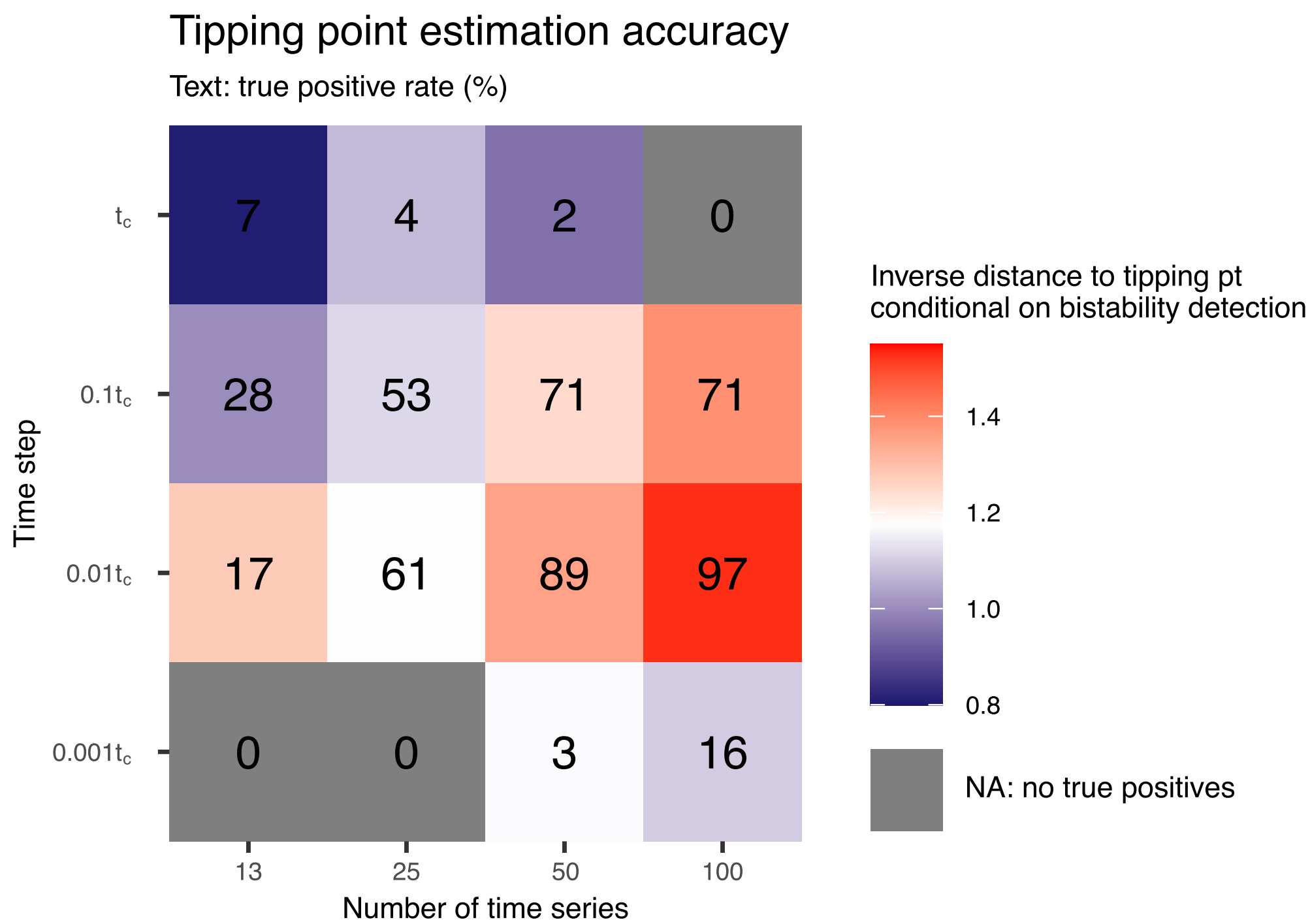

Figure S3 Estimation accuracy of tipping point location depending on the time step and number of short time series. For every bistable simulation in Figure 3f for which the model correctly inferred bistability, we calculated the mean distance to the tipping point for each of the bistable posterior draws. The color of each cell represents the inverse mean of these distances over the correctly recovered bistable simulations, the number of which is written on the cell. This quantity could not be calculated for cells where no simulation out of 100 was inferred as bistable.

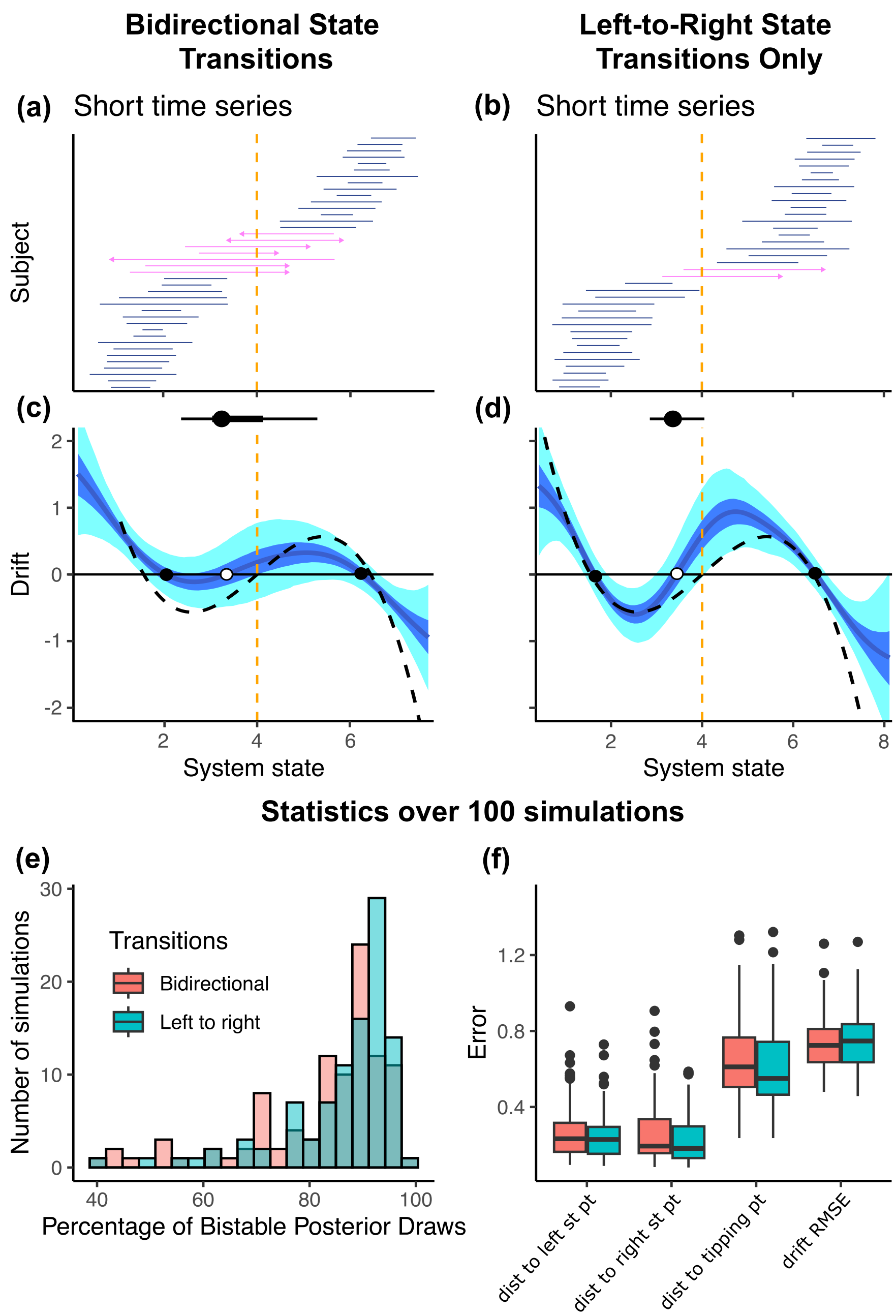

Figure S4 Model performance with state-transition bias. (a) A set of short time series simulated from the CUSP model with 5 time points each separated by $0.01t_c$. The ground truth drift is symmetric with equally stable stable states on either side of the tipping point. Pink time series represent transitions across the tipping point (orange) and the arrow indicates which basin the trajectory transitioned into. In this case, transitions occurred in both directions. (b) The same number of time series were simulated as in (a) but only the transitions that occurred from the left basin to the right basin were kept. (c) The drift function output by the model along with 50% and 95% credible intervals. The dashed line is the ground truth drift function and above it the tipping region represented by its mean (point) and 95% credible interval. The inferred stable states of the posterior mean are represented by black points and the tipping point as a white point. (d) The drift function inferred from the biased time series in (b) with the same ground truth drift function as in (c). (e) For both the biased and unbiased cases, we collected statistics over 100 simulations with the same underlying system. For each simulation, we recorded the percentage of posterior draws that were bistable. (f) From the draws that were bistable, we calculated the mean distance to the stable state in the left basin, the right basin, the tipping point, and the mean Root Mean Square Error (RMSE) between the ground truth drift function and the bistable posterior draws.

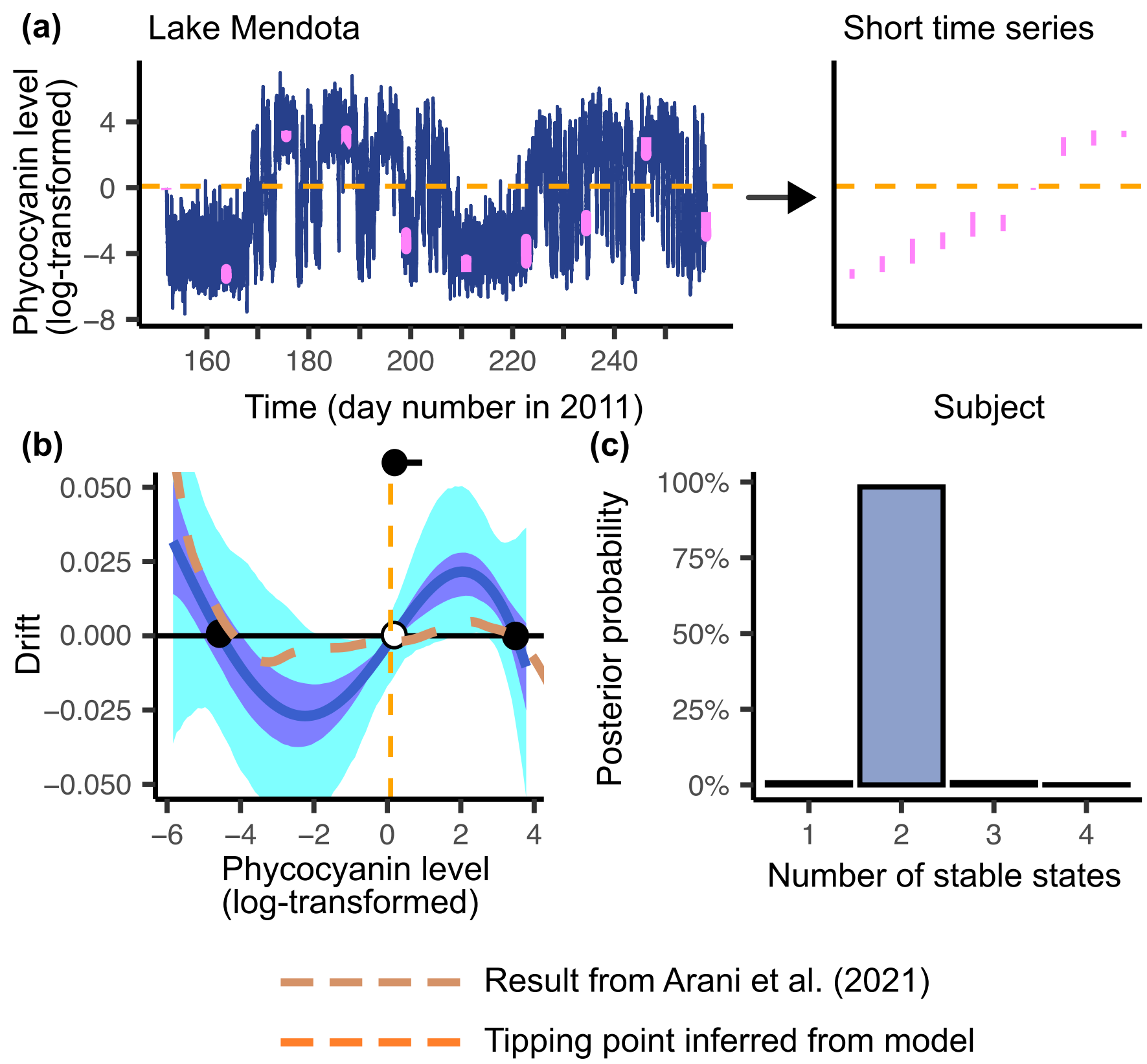

Figure S5 Re-analysis of Lake Mendota data with fewer time points. (a) A recent study on Lake Mendota [14] used 60,000 equally spaced time points (left). We selected a small subset of 50 time points evenly distributed over the data range to simulate collection of short and sparse time series (right); the vertical pink lines represent these 10 short time series of 5 points. The dashed orange line the tipping point inferred by our model. (b) With only 50 time points, the drift function result from Arani et el. (2021) (dashed brown line) fell within the 95% credible interval of our model using three orders of magnitude fewer time points (5×10^1 compared to 6×10^4). Shown in the upper margin is the tipping region. (c) Corresponding multistability posterior. Our Bayesian formulation provides 98% support for bistability in this ecosystem, in line with previous results. This outlines the model's ability to accurately identify stability characteristics including the locations of the stable modes with very few time points. Nevertheless, the stability of the diffusion estimate greatly decreases (not shown).

The systems studied & the 62 most prevalent taxa selected for analysis

System	Stability	Exit Time	System	Stability	Exit Time
CUSP Model Simulations	bistable	calculated	Escherichia_coli_et_rel	unistable	not calculated
Custom SDE Model Simulations	unistable	not calculated	Eubacterium_biforme_et_rel	bistable	calculated
Lake Mendota	bistable	calculated	Eubacterium_hallii_et_rel	unistable	not calculated
Allistipes_et_rel	unistable	not calculated	Eubacterium_rectale_et_rel	unistable	not calculated
Anaerostipes_caccae_et_rel	unistable	not calculated	Eubacterium_ventriosum_et_rel	unistable	not calculated
Anaerotruncus_colihominis_et_rel	unistable	not calculated	Faecalibacterium_prausnitzii_et_rel	unistable	not calculated
Anaerovorax_odorimutans_et_rel	unistable	not calculated	Lachnobacillus_bovis_et_rel	unistable	not calculated
Bacteroides_fragilis_et_rel	unistable	not calculated	Lachnospira_pectinoschiza_et_rel	unistable	not calculated
Bacteroides_ovatus_et_rel	unistable	not calculated	Lactobacillus_gasseri_et_rel	unistable	not calculated
Bacteroides_plebeius_et_rel	unistable	not calculated	Lactobacillus_plantarum_et_rel	unistable	not calculated
Bacteroides_splachnicus_et_rel	unistable	not calculated	Oscillospira_guillermondii_et_rel	unistable	not calculated
Bacteroides_stercoris_et_rel	unistable	not calculated	Outgrouping_clostridium_cluster_XIVa	unistable	not calculated
Bacteroides_uniformis_et_rel	unistable	not calculated	Oxalobacter_formigenes_et_rel	unistable	not calculated
Bacteroides_vulgatus_et_rel	unistable	not calculated	Papillibacter_cinnamivorans_et_rel	unistable	not calculated
Bifidobacterium	bistable	calculated	Parabacteroides_distasonis_et_rel	unistable	not calculated
Bryantella_formatexigens_et_rel	unistable	not calculated	Prevotella_melaninogenica_et_rel	bistable	calculated
Butyrivibrio_crossotus_et_rel	unistable	not calculated	Prevotella_tannerae_et_rel	unistable	not calculated
Clostridium_cellulosi_et_rel	unistable	not calculated	Roseburia_intestinalis_et_rel	unistable	not calculated
Clostridium_colinum_et_rel	unistable	not calculated	Ruminococcus_bromii_et_rel	unistable	not calculated
Clostridium_difficile_et_rel	unistable	not calculated	Ruminococcus_callidus_et_rel	unistable	not calculated
Clostridium_leptum_et_rel	unistable	not calculated	Ruminococcus_gnavus_et_rel	unistable	not calculated
Clostridium_nexile_et_rel	unistable	not calculated	Ruminococcus_lactaris_et_rel	unistable	not calculated
Clostridium_orbiscindens_et_rel	unistable	not calculated	Ruminococcus_obeum_et_rel	unistable	not calculated
Clostridium_sensu_stricto	unistable	not calculated	Sporobacter_termitidis_et_rel	unistable	not calculated
Clostridium_sphenoides_et_rel	unistable	not calculated	Streptococcus_bovis_et_rel	unistable	not calculated
Clostridium_stercorarium_et_rel	unistable	not calculated	Streptococcus_mitis_et_rel	unistable	not calculated
Clostridium_symbiosum_et_rel	unistable	not calculated	Subdoligranulum_variable_at_rel	unistable	not calculated
Collinsella	unistable	not calculated	Sutterella_wadsworthia_et_rel	unistable	not calculated
Coprobacillus_catenaformis_et_rel	unistable	not calculated	Tannerella_et_rel	unistable	not calculated
Coprococcus_eutactus_et_rel	unistable	not calculated	Uncultured_Clostridiales_I	unistable	not calculated
Dialister	unistable	not calculated	Uncultured_Clostridiales_II	unistable	not calculated
Dorea_formicigenerans_et_rel	unistable	not calculated	Uncultured_Mollicutes	bistable	calculated
Enterobacter_aerogenes_et_rel	unistable	not calculated			

Figure S6 An overview of the systems analyzed with our model including their stability properties and whether the exit time for that system was calculated. Systems that were not a part of the HITChip dataset are coloured pink. Only the 62 most prevalent taxa in the HITChip dataset were studied. For all HITChip bacterial taxa, we used a cut-off of 45 days for the time between observations, which corresponds to time steps ranging from $0.01t_c$ to $0.06t_c$ depending on taxon. This allowed us to use the same number of time points for each taxon. As exit time requires local stability, only systems that were classified as bistable by the model were eligible for exit time calculation.

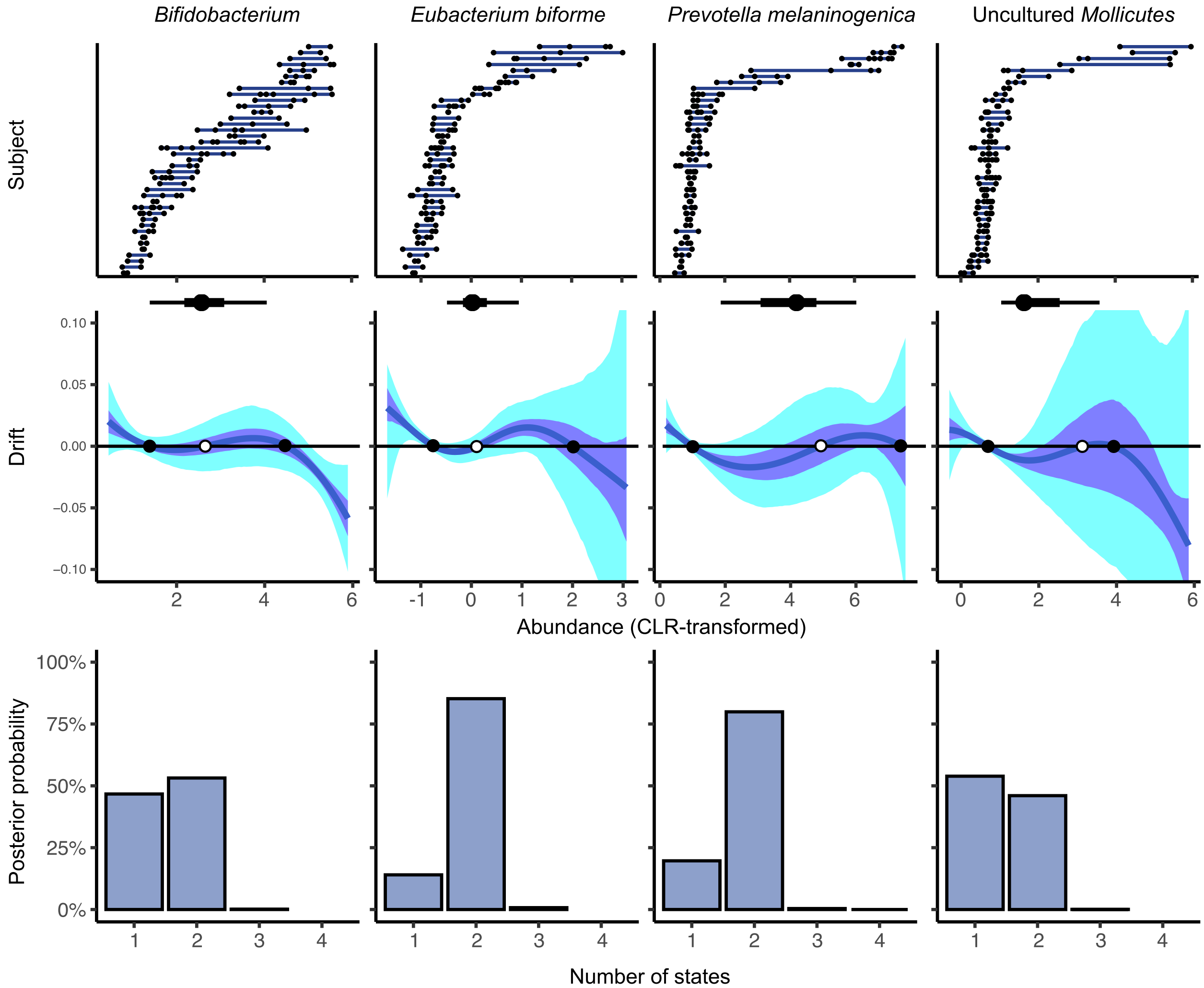

Figure S7 Four taxa in the HITChip data set that show evidence of bistability. Each time series (upper row) consists of 2-6 time points for 39 subjects (total of 127 points). The posterior drift outputs with 50% and 95% credible intervals enveloping the means, including the stable modes (black points), tipping points (white circle) (middle row), and tipping regions in the top margins with their own means and 50% and 95% credible intervals (top whisker plots). Corresponding multistability posteriors (bottom row).

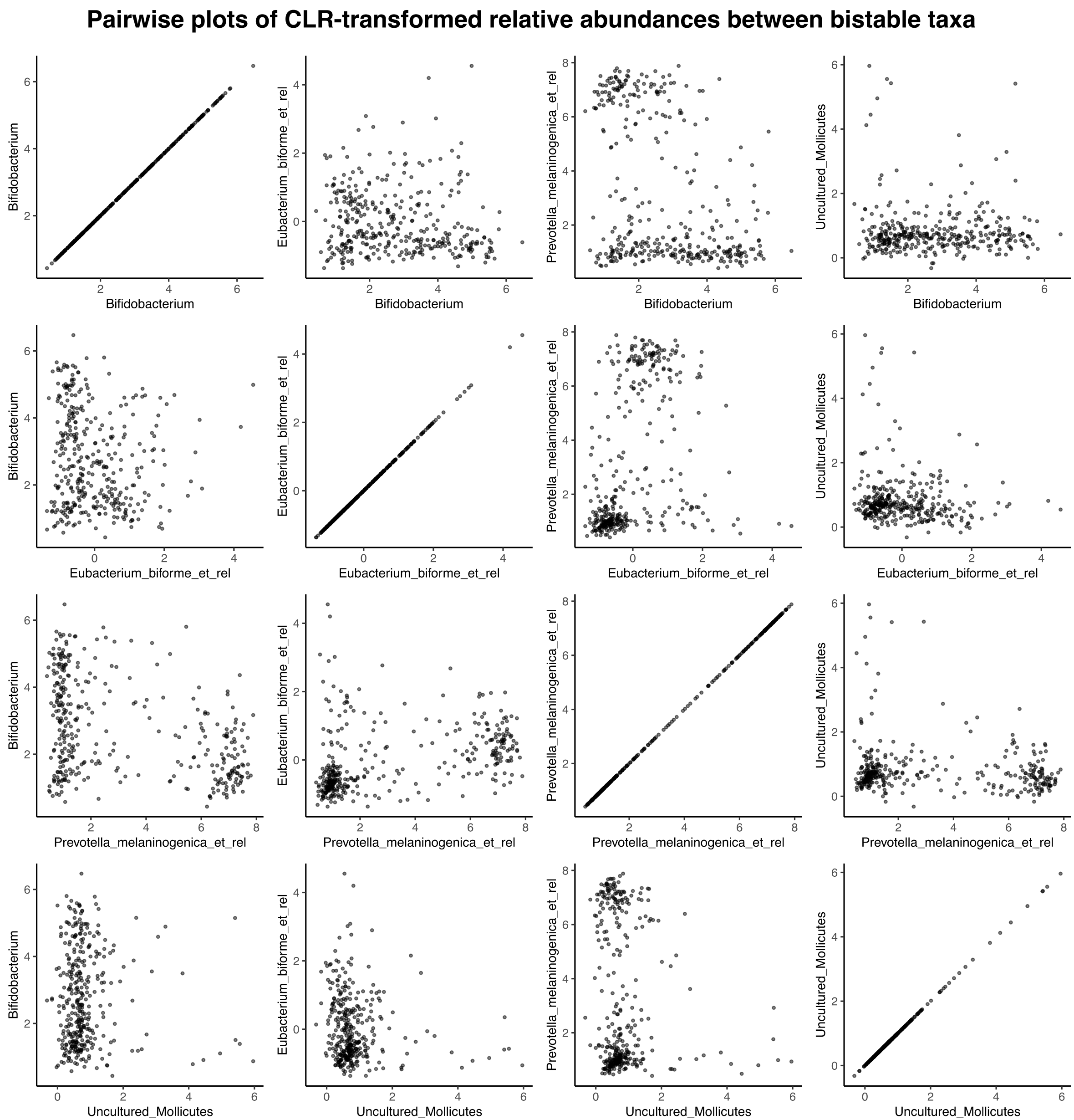

Figure S8 Pairwise relationships between the four taxa identified as bistable by the model. Each panel shows the CLR-transformed relative abundances of a pair of taxa across samples. In several cases, the bimodal abundance structure of one taxon is largely independent of the abundance of the other, indicating weak coupling between their stable states, such as with *Bifidobacterium* and *Prevotella melaninogenica*. We also observe patterns consistent with partial exclusion, where high abundance of one taxon tends to be found when the other has low abundance, such as *Eubacterium biforme* and *Uncultured Mollicutes*.

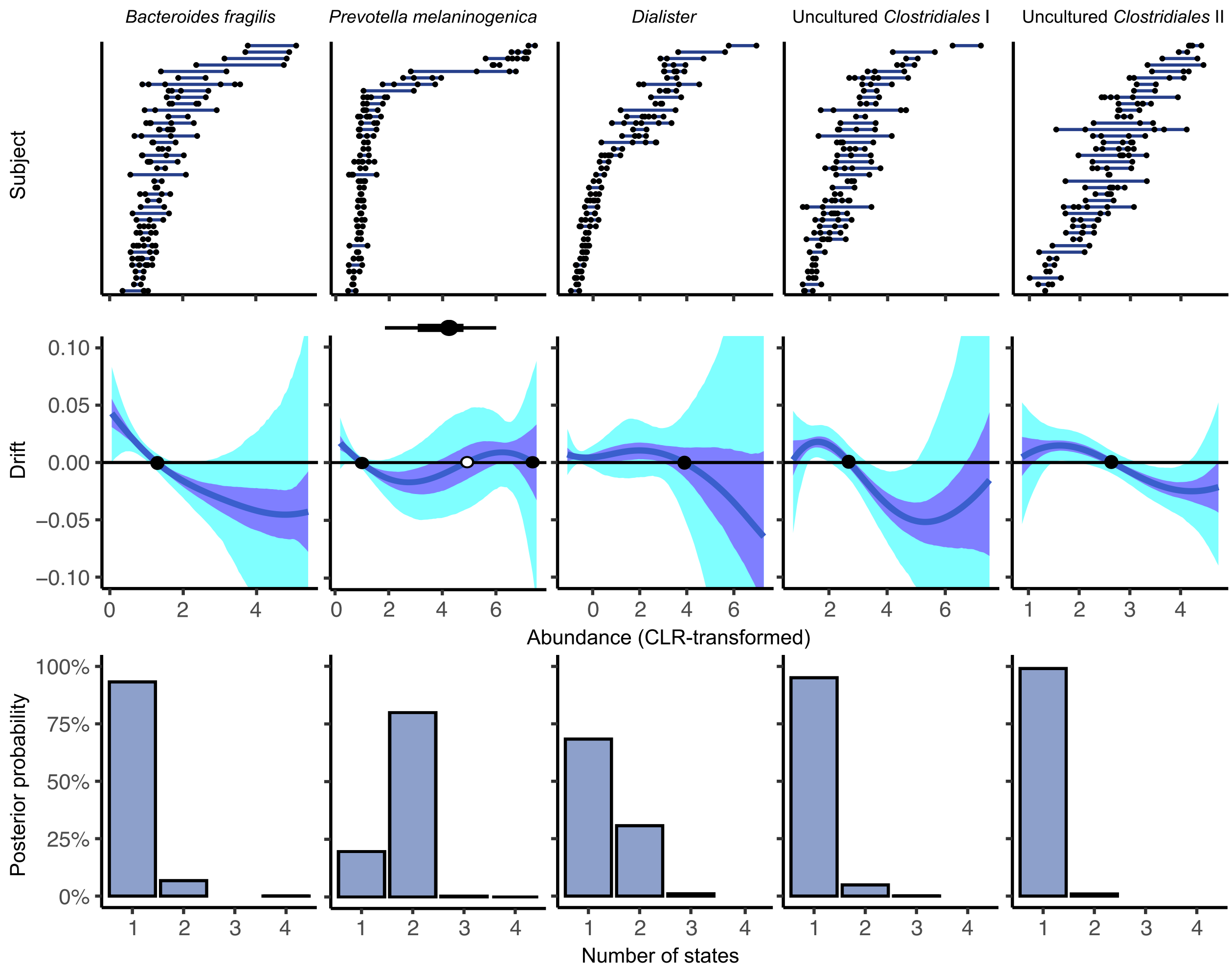

Figure S9 The six taxa identified as tipping elements in Lahti et al. (2014). Each time series (upper row) consists of 2-6 time points for 39 subjects (total of 127 points). The posterior drift outputs with 50% and 95% credible intervals enveloping the means, including the stable modes (black points), tipping points (white circle) (middle row), and tipping regions in the top margins with their own means and 50% and 95% credible intervals. Corresponding multistability posteriors (bottom row).

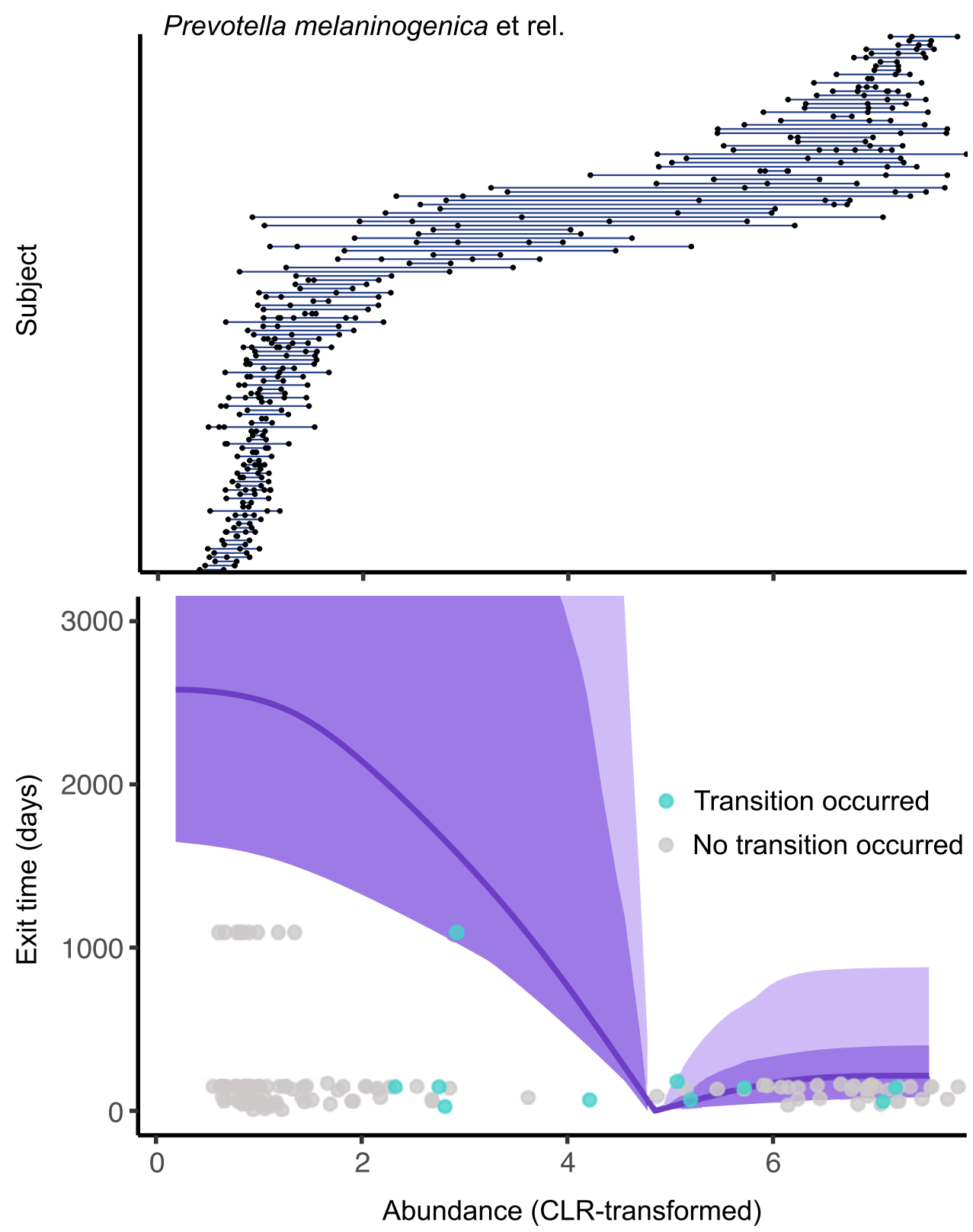

Figure S10 Transition time scales present in the gut microbiota. The time series from the 128 healthy adult subjects (2-6 time points each, 358 in total) across all time steps in the HITChip data set for *Prevotella melaninogenica* et rel. (upper). A zoomed-in version of Fig. 5b. Grey points represent short time series that started at the given state value and did not transition within the given time and cyan points represent those that did. This shows that the exit time as a function of the abundance is on the same order of magnitude as the transition time scales obtained from directly from the data.
